# The Efficacy and Safety of *Helicobacter pylori* Eradication Regimens in Patients Receiving Hemodialysis: A Systematic Review and Network Meta-Analysis

**DOI:** 10.1159/dig/acmag012

**Published:** 2026-09-05

**Authors:** Satoshi Sato, Jun Watanabe, Takeshi Kanno, Keigo Misawa, Hiromi Sekiguchi, Tomonori Yano, Atsushi Masamune, Tomoari Kamada

**Affiliations:** a Division of Gastroenterology, Department of Medicine, Jichi Medical University, Shimotsuke, Japan; b Division of Gastroenterological, Department of Surgery, General and Transplant Surgery, Jichi Medical University, Shimotsuke, Japan; c Division of Community and Family Medicine, Jichi Medical University, Shimotsuke, Japan; d Division of Gastroenterology, Department of Medicine, Farncombe Family Digestive Health Research Institute, McMaster University, Hamilton, ON, Canada; e Division of Gastroenterology, Tohoku University Graduate School of Medicine, Sendai, Japan; f R & D Division of Career Education for Medical Professionals, Medical Education Center, Jichi Medical University, Shimotsuke, Japan; g Department of Health Care Medicine, Kawasaki Medical School General Medical Center, Okayama, Japan

**Keywords:** *Helicobacter pylori* eradication, Hemodialysis, Network meta-analysis

## Abstract

**Introduction:**

*Helicobacter pylori* eradication is clinically important in patients on hemodialysis (HD), who are at risk for serious gastrointestinal complications. However, altered drug metabolism and antibiotic resistance in patients on HD complicate *H. pylori* eradication. This study aimed to perform a network meta-analysis to synthesize existing evidence on the efficacy and safety of *H. pylori* eradication regimens in patients on HD.

**Methods:**

We searched MEDLINE, the Cochrane Library, EMBASE, Ichushi-Web, ICTRP, and ClinicalTrials.gov. Two reviewers independently selected randomized controlled trials that evaluated *H. pylori* eradication therapy in patients undergoing HD. We performed a frequentist random effects network meta-analysis. The primary outcome was eradication rate, and the secondary outcomes were treatment completion rates and all adverse events. We used CINeMA to assess the confidence in the evidence. The study protocol was registered at OSF (https://osf.io/gn978/).

**Results:**

Ten trials involving 481 patients were included in the analysis. Seven eradication regimens were compared: six concomitant and one sequential regimen. Relative to standard-dose proton pump inhibitor/clarithromycin/amoxicillin regimens (PAC), the evidence is very uncertain about the effect of the proton pump inhibitor/clarithromycin regimen on the eradication rate (odds ratio, 0.11; 95% confidence interval, 0.01–1.07). For reduced-dose PAC (in which clarithromycin or amoxicillin is dose reduced), alternative regimens (with azithromycin, metronidazole, or levofloxacin), and sequential regimens, the estimates were too imprecise to determine whether a difference from PAC exists (low certainty). Treatment completion rates were generally favorable (83–100%), and adverse events were most frequent with standard-dose PAC (45%).

**Conclusion:**

Reduced-dose PAC and alternative and sequential regimens may be considered for *H. pylori* eradication in patients on HD.

## Introduction

*Helicobacter pylori* infection is a known risk factor for several diseases, including peptic ulcers and gastric cancer, and eradication therapy is strongly recommended. The prevalence of *H. pylori* infection among patients undergoing hemodialysis (HD) is lower than that in the general population. Nonetheless, 27.5–48.6% of such patients remain *H. pylori*-positive [1–3]. Notably, *H. pylori* eradication therapy in this population is key for preventing substantial gastrointestinal complications, particularly peptic ulcer recurrence and rebleeding. Some studies have demonstrated that patients on HD with acute nonvariceal upper gastrointestinal bleeding have 30-day mortality rates of 10.7–13.7% [4, 5], which are higher than that for non-HD patients. *H. pylori* eradication significantly reduces peptic ulcer recurrence rates in patients with *H. pylori* positivity [6]. Therefore, *H. pylori* eradication in patients on HD is important for preventing upper gastrointestinal bleeding.

Altered drug metabolism and rising antibiotic resistance make *H. pylori* eradication particularly challenging in patients receiving HD [7, 8]; thus, identifying an effective *H. pylori* eradication regimen in this population is clinically important. For example, amoxicillin, a key drug in standard *H. pylori* eradication regimens, has a markedly prolonged half-life in patients on HD, and a single 250-mg dose administered after HD provides adequate serum levels for 24 h [7]. A meta-analysis demonstrated that reducing the amoxicillin dose by 1/2–1/3 in the proton pump inhibitor/clarithromycin/amoxicillin (PAC) regimen can prevent adverse events while maintaining an eradication rate similar to that in the general population [2]. The latest guidelines from the Japanese Society for Helicobacter Research recommend a PAC regimen with a reduced amoxicillin dose for *H. pylori* eradication in patients receiving HD (evidence level C) [9]. However, the optimal *H. pylori* eradication regimen for patients undergoing HD remains unknown, as few studies have compared regimens or synthesized the evidence in this population. A network meta-analysis efficiently synthesizes both direct and indirect evidence and enables a more complete evaluation of efficacy and safety for each regimen. This study aimed to perform a network meta-analysis integrating existing evidence on eradication in patients on HD to enable a more comprehensive comparative assessment of treatment strategies.

## Methods

This study was performed in accordance with the Preferred Reporting Items for Systematic Review and Meta-Analysis 2020 (PRISMA-2020) and PRISMA for Network Meta-Analyses (PRISMA-NMA) [10, 11] (the PRISMA checklist and PRISMA-NMA checklist are given in online suppl. Information 1; for all online suppl. material, see https://doi.org/10.1159/dig/acmag012). The study protocol was registered at OSF (https://osf.io/gn978/).

### Study Selection

Randomized controlled trials (RCTs) assessing efficacy and adverse events associated with *H. pylori* eradication in patients on HD were included. No restrictions were applied regarding language or country. All records were considered, including published and unpublished articles, conference abstracts, and letters. Non-RCTs were excluded. Moreover, research was not excluded based on observation period or publication year. Studies enrolling both patients on and off HD were included when outcome data specific to the HD subgroup were available. If these data were unavailable, the studies were included in the primary analysis but excluded from the sensitivity analysis.

The primary outcome was the eradication rate for each regimen. Secondary outcomes included treatment completion rate and all adverse events. Treatment completion rates were calculated using the intention-to-treat (ITT) principle. Detailed definitions of study outcomes are provided in the online supplementary Information.

### Types of Interventions

For the network meta-analysis, treatment regimens were predefined and categorized as concomitant therapy (fixed combination throughout treatment) or sequential therapy (stepwise antibiotic changes during treatment). Detailed definitions of each regimen are summarized in the online supplementary Information.

Concomitant therapy included the following regimens:

- Standard-dose PAC (regimen containing amoxicillin at standard doses)
- Reduced-dose PAC (rPAC) (regimens containing amoxicillin at reduced doses)
- Metronidazole-containing regimens
- Proton pump inhibitor/amoxicillin/azithromycin (PA+AZM)
- Proton pump inhibitor/amoxicillin/levofloxacin (PA+LVFX)
- Proton pump inhibitor/clarithromycin (PC)

Sequential therapy (seq) included regimens with stepwise changes in antibiotics during treatment.

### Data Source

MEDLINE (PubMed), the Cochrane Central Register of Controlled Trials (Cochrane Library), EMBASE (Dialog), and Ichushi-Web (Japan Medical Abstracts Society) were searched without limitations regarding publication year or language. Ongoing or unpublished trials were also searched in the World Health Organization International Clinical Trials Platform Search Portal and ClinicalTrials.gov. Reference lists, cited articles describing eligible studies, and international guidelines were checked [9, 12–14]. All databases were searched on August 30, 2025. Authors of the original studies were contacted for unpublished or additional data. The search strategy is summarized in online supplementary Information.

### Screening, Data Collection, and Risk of Bias Assessment

Two independent reviewers (S.S. and J.W.) screened titles and abstracts, assessed eligibility based on full texts, and extracted data. Disagreements were resolved by discussion or, when needed, consultation with an additional investigator (T.K.). Extracted data included the study design, study population, interventions, and outcomes, and the study region was prespecified as an effect modifier. Two of the three reviewers (S.S. and J.W.) independently assessed risk of bias at the outcome level using the Risk of Bias 2 tool [15]. The effect of assignment to the intervention (intention-to-treat effect) was evaluated [16]. Any unresolved discrepancies were adjudicated by T.K.

### Data Synthesis and Statistical Analysis

Odds ratios (ORs) and 95% confidence intervals (CIs) comparing PAC regimens with other regimens were pooled for eradication rates, a binary outcome. Treatment completion and adverse events were summarized descriptively. The feasibility of quantitative synthesis was assessed by considering the sparsity of events and, particularly for adverse events, the comparability of definitions, ascertainment methods, and reporting units across trials, in accordance with the Cochrane Handbook. Group-level data were used. Additionally, binomial likelihood was applied for dichotomous outcomes and normal likelihood for continuous outcomes. Using a random-effects frequentist network meta-analysis model, study effect sizes were synthesized. Correlations induced by multi-arm studies were addressed using multivariate distributions. Heterogeneity variance (variance of the random-effects distribution) was used to quantify across-study and within-comparison variability in treatment effects. The analysis was performed using MetaInsight (version 6.4.0) [17].

Clinical trial registry systems (ClinicalTrials.gov and ICTRP) were searched, and an extensive literature search was conducted for unpublished trials. Outcome reporting bias was assessed by comparing outcomes specified in trial protocols with those reported in publications. Publication bias was evaluated by visual inspection of funnel plots. Nonetheless, Egger’s test was not performed because only 10 trials were included. To assess the robustness of the results, we performed sensitivity analyses of the primary outcomes by conducting per-protocol analyses, excluding studies that included non-HD patients and excluding the single trial contributing the PC arm.

Potential effect modifiers included study regions because antibiotic resistance rates differ across geographic areas [18]. These variables were checked for similar distribution across studies grouped by comparison. Meta-regression was not performed because the limited number of studies precluded reliable estimation of covariate effects. Transitivity was assessed descriptively by examining the distribution of study countries across treatment comparisons. If a violation of the transitivity assumption was suspected, only pairwise MA, rather than NMA, was conducted.

Confidence in each primary outcome was assessed by two reviewers (S.S. and J.W.) using the CINeMA tool [19]. The CINeMA framework comprises within- and across-study bias, indirectness, imprecision, heterogeneity, and incoherence. For within-study bias and indirectness, CINeMA calculates each study’s contribution to each network estimate and integrates these contributions with study-specific ratings (low, moderate, and high) to grade relative effects for each network comparison.

## Results

Figure 1 depicts the data inclusion process. The search yielded 111 unique records after duplicate removal and article review. Following title and abstract screening, 10 studies including 481 participants were identified as RCTs that compared regimen efficacy for *H. pylori* eradication in patients [20–29].

**Fig. 1.**
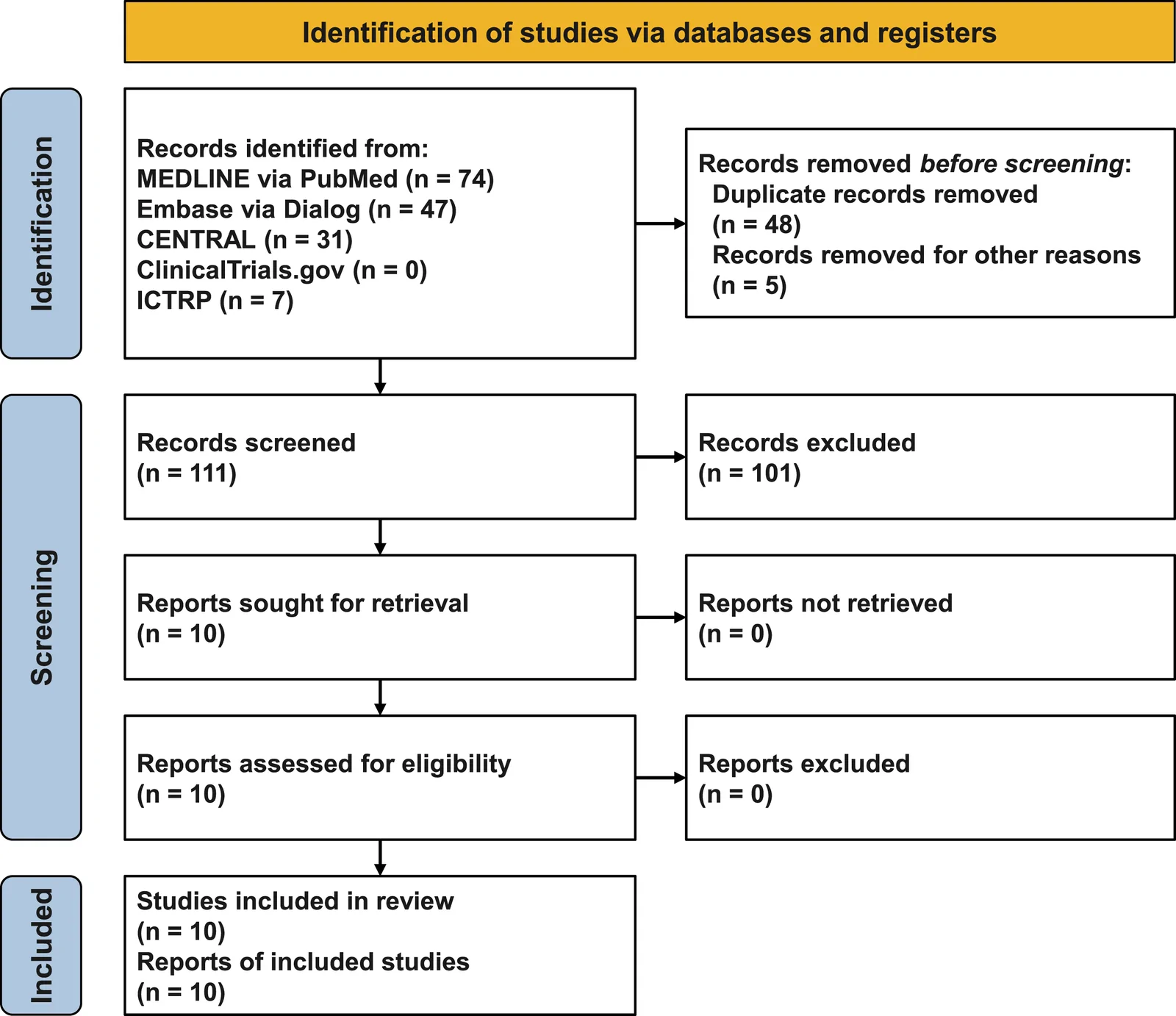
Flow of the study selection processes.

Table 1 summarizes the characteristics of the included studies. Across the 10 RCTs, age and body mass index were generally comparable. In studies reporting time from dialysis initiation, all patients had received maintenance dialysis for ≥3 months. All participants receiving HD remained clinically stable throughout the study period.

**Table 1.** Characteristics of the included studies

Authors [ref no.]	Year	Country	Group	Number of patients	Age (years)	Male	Female	BMI mean	Dialysis duration (months)	Assessment of eradication method/duration
Simunic, et al. [20]	2005	Croatia	All	47	NR	NR	NR	NR	NR	Biopsy, RUT/4w
			MNZ	23	NR	NR	NR	NR		
			PA+AZM	24	NR	NR	NR	NR		
Itatsu, et al. [21]	2007	Japan	All	20	65.8	NR	NR	NR	53.3±52.6	Biopsy/12w
			rPAC^1^	11	NR	NR	NR	NR		
			PC	9	NR	NR	NR	NR		
Seyyedmajid, et al. [22]	2011	Iran	All	40	52.1	NR	NR	23.7	NR	UBT/6w
			rPAC^1^	20	NR	NR	NR	NR		
			Seq	20	NR	NR	NR	NR		
Jalazadeh, et al. [23]	2012	Iran	All	39	59	20	19	24.6	40.26±34.8	fAg/8w
			rPAC^1^	19	59	11	8	24.4		
			PA+AZM	20	59	9	11	24.8		
Ehsani, et al. [24]	2014	Iran	All	66	47.5	28	38	25.1	39.43±29.7	fAg UBT/4w
			PAC	35	46.2	14	21	24.7		
			rPAC^1^	31	49	14	17	25.6		
Makhlough, et al. [25]	2014	Iran	All	28	NR	NR	NR	26.4	≥3	fAg/8w
			rPAC^1^	16	NR	NR	NR	NR		
			Seq	12	NR	NR	NR	NR		
Makhlough, et al. [26]	2016	Iran	All	40	51	19	21	NR	NR	fAg/8w
			rPAC^1^	20	51	11	9	NR		
			Seq	20	51.5	8	12	NR		
Alirezaei, et al. [27]	2017	Iran	All	44	45.5	29	15	24.3	NR	fAg, UBT/4w
			PAC	22	45.2	14	8	23.8		
			PA+LVFX	22	46.3	15	7	23.2		
Sahara, et al. [28]	2018	Japan	All	37	66.2	25	12	21.5	51.6±45.6	UBT/8w
			PAC	18	67	12	6	21.8		
			rPAC^1^	19	65.5	13	6	21.1		
Seyyedmajid, et al. [29]	2018	Iran	All	120	43.1	67	53	24.6	NR	UBT/6w
			rPAC^1^	30	43.9	17	13	24.2		
			MNZ	60	43.4	34	26	NR		
			Seq	30	41.7	16	14	24.5		

A, amoxicillin; AZM, azithromycin; C, clarithromycin; fAg, fecal antigen test; LVFX, levofloxacin; MNZ, metronidazole; NR, not reported; P, proton pump inhibitor; RUT, rapid urease test; Seq, sequential therapy; UBT, urea breath test.

^1^ rPAC: PAC regimen with reduced dose of amoxicillin.

Across all randomized controlled trials, treatment regimens were evaluated in the following numbers of trials: PAC (three studies), rPAC (eight studies), PC (one study), PA+ LVFX (one study), PA + AZM (two studies), metronidazole regimens (two studies), and seq (four studies). Twenty-one treatment groups were included because one study [29] had three groups (shown in Fig. 2). Overall, the risk of bias was rated as some concerns in nine trials and low in one trial; no trial was rated at high risk of bias (online suppl. Fig.1). The contribution of each study to the network estimates, weighted by its risk-of-bias rating, is shown in online supplementary Fig. 2.

**Fig. 2.**
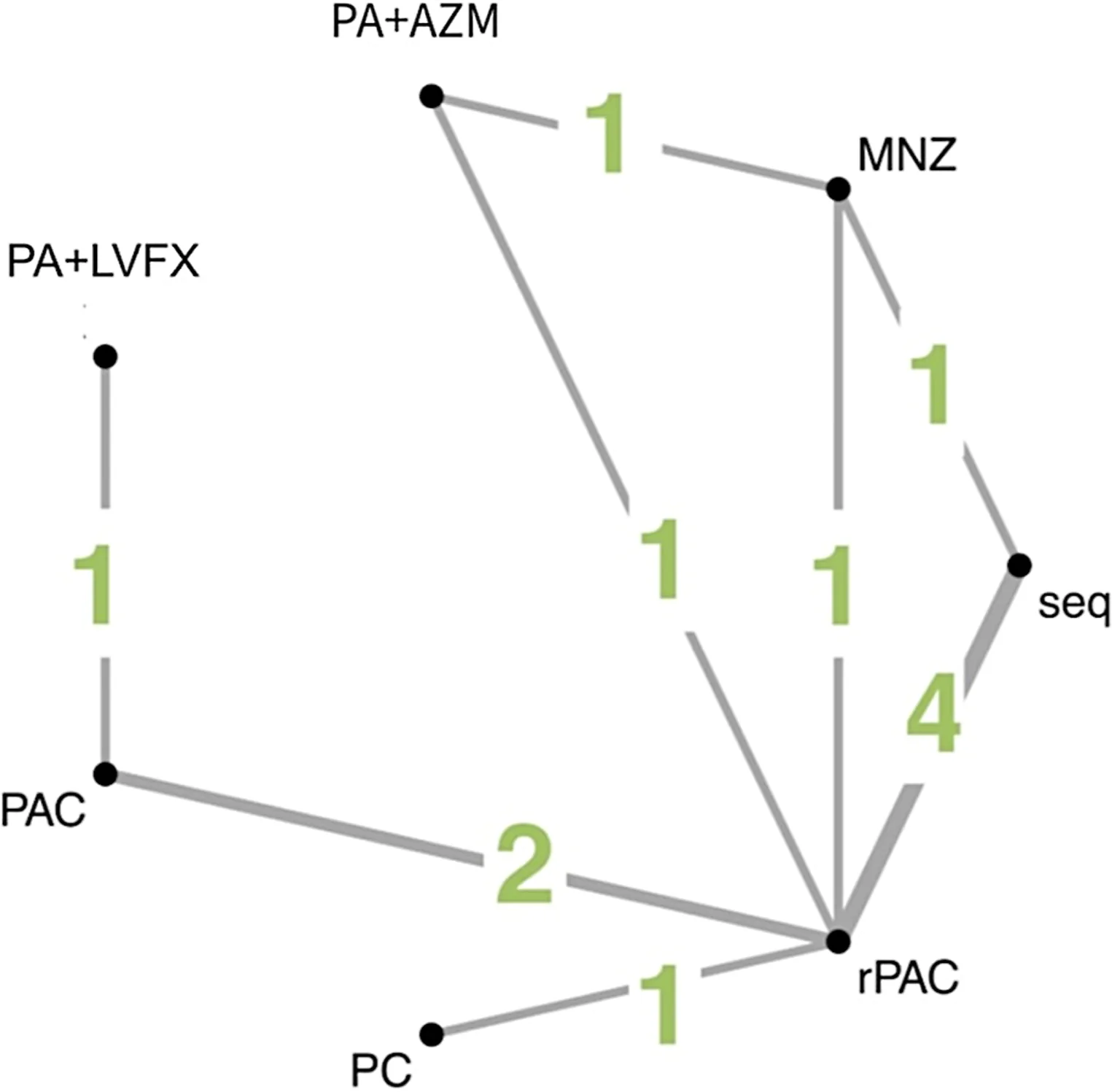
Network plot for the eradication rate in all regimens. The thickness of these lines corresponds to the amount of evidence in a single comparison. PAC, proton pump inhibitor/clarithromycin/amoxicillin; rPAC, PAC with reduced dose of clarithromycin or amoxicillin; MNZ, metronidazole-containing regimens; PA+AZM, proton pump inhibitor/amoxicillin/azithromycin; PA+LVFX, proton pump inhibitor/amoxicillin/levofloxacin; PC, proton pump inhibitor/clarithromycin; seq, sequential therapy.

Transitivity was assessed via descriptive comparisons of study characteristics. No major concerns regarding transitivity were identified based on available study-level characteristics. Based on existing evidence [30–34], antimicrobial-resistance patterns were broadly comparable across countries for regimens included in this network. Therefore, no clinically meaningful imbalance in this effect modifier was identified, supporting the plausibility of the transitivity assumption.

Global inconsistency was assessed using a random-effects design-by-treatment interaction model. No statistical evidence of global inconsistency was observed (χ^2^ = 0.208, df = 2; *p* = 0.901). Within the CINeMA framework, the incoherence domain raised no concerns because no important disagreements between direct and indirect evidence were identified.

### Primary Outcome

#### Eradication Rate (ITT)

Ten trials involving 481 patients were included. Table 2 summarizes confidence in network estimates for eradication rates. The evidence is very uncertain about the effect of the PC regimen on the *H. pylori* eradication rate relative to PAC (odds ratio [OR] 0.11; 95% CI: 0.01–1.07). For the remaining regimens, the estimates were too imprecise to determine whether a difference from PAC exists (low certainty; Fig. 3). The league table for all pairwise comparisons is provided in online supplementary Table 1.

**Table 2.** Summary of confidence in network estimates for eradication rate

Comparison (vs. PAC)	Number of studies	Within-study bias	Reporting bias	Indirectness	Imprecision	Heterogeneity	Incoherence	Confidence rating
rPAC^1^	2	Some concerns	Low risk	No concerns	Major concerns	No concerns	No concerns	Low
PA+LVFX	1	Some concerns	Low risk	No concerns	Major concerns	No concerns	No concerns	Low
MNZ	0	Some concerns	Low risk	No concerns	Major concerns	No concerns	No concerns	Low
PA+AZM	0	Some concerns	Low risk	No concerns	Major concerns	No concerns	No concerns	Low
PC	0	Some concerns	Some concerns	No concerns	Major concerns	No concerns	No concerns	Very low
Seq	0	Some concerns	Low risk	No concerns	Major concerns	No concerns	No concerns	Low

A, amoxicillin; AZM, azithromycin; C, clarithromycin; LVFX, levofloxacin; MNZ, metronidazole; P, proton pump inhibitor; Seq, sequential therapy.

^1^ rPAC: PAC regimen with reduced dose of amoxicillin.

**Fig. 3.**
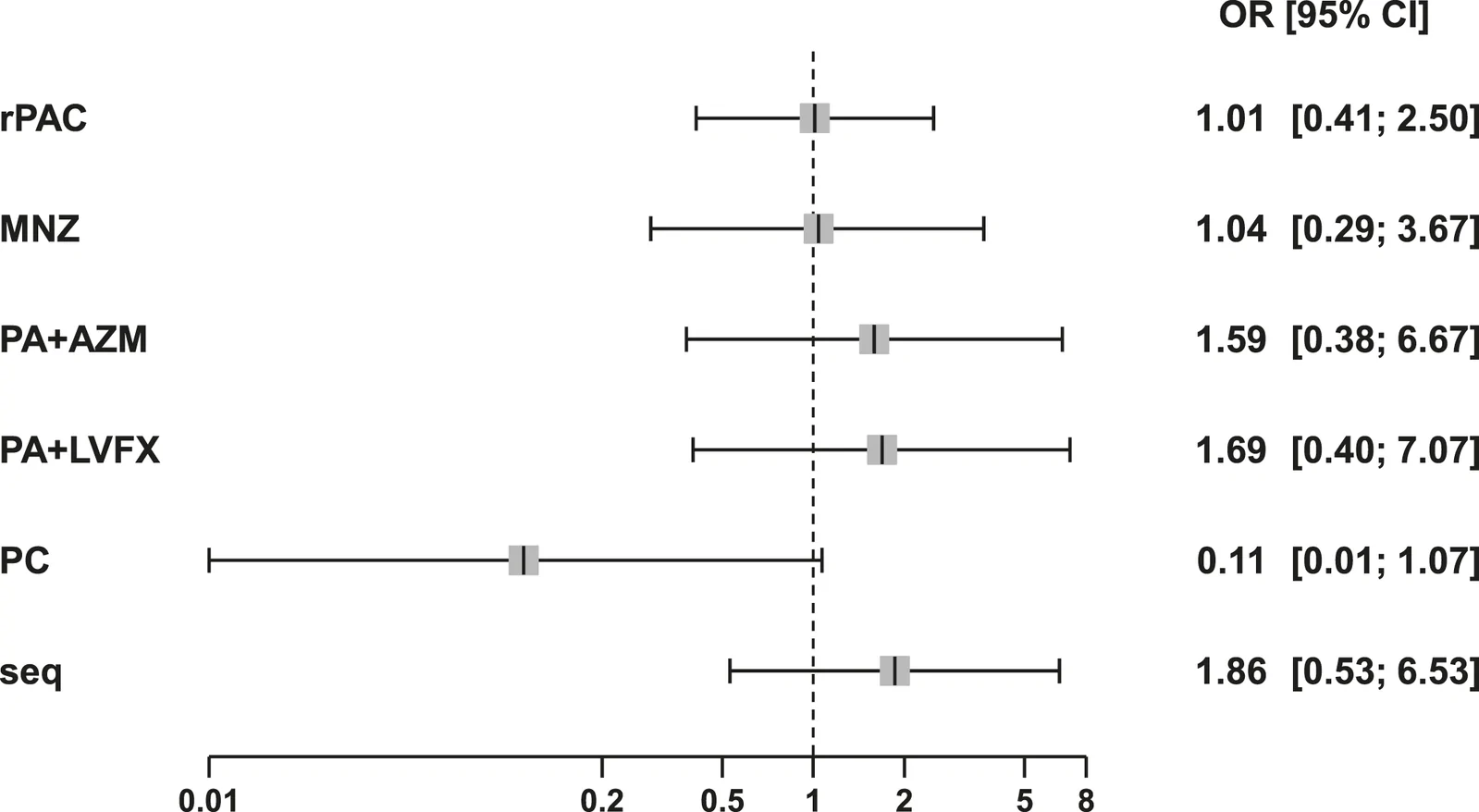
Eradication rate of each regimen compared with the PAC regimen in random effects and Frequentist network meta-analysis (intention to treat). OR, odds ratio; CI, confidence interval.

### Secondary Outcomes

#### Treatment Completion Rates

The treatment completion rates in each study are listed in Table 3. Treatment completion rates were generally favorable across all studies, ranging from 83% to 100% in the ITT population.

**Table 3.** Treatment completion rates in each study

Study	Group	Patients, *n*		Treatment completed patients (*n*)	Treatment completion rate (%)	
		mITT^1^	ITT		mITT^1^	ITT
Šimunić et al. [20] (2005)	MNZ	21	23	21	100	91
	PA+AZM	23	24	23	100	96
Itatsu et al. [21] (2007)	rPAC^2^	111	11	11	100	100
	PC	9	9	8	89	89
Seyyedmajid et al. [22] (2011)	rPAC^2^	20	20	20	100	100
	Seq	20	20	19	95	100
Jalazadeh et al. [23] (2012)	rPAC^2^	17	19	17	100	89
	PA+AZM	20	20	20	100	100
Ehsani Ardakani et al. [24] (2014)	PAC	35	35	35	100	100
	rPAC^2^	31	31	31	100	100
Makhlough et al. [25] (2014)	rPAC^2^	15	16	15	100	94
	Seq	11	12	10	91	83
Makhlough et al. [26] (2016)	rPAC^2^	20	20	20	100	100
	Seq	20	20	20	100	100
Alirezaei et al. [27] (2017)	PAC	22	22	22	100	100
	PA+LVFX	22	22	22	100	100
Sahara et al. [28] (2018)	PAC	18	18	18	100	100
	rPAC^2^	18	19	18	100	95
Seyyed et al. [29] (2018)	rPAC^2^	30	30	30	100	100
	MNZ	60	60	60	100	100
	Seq	30	30	30	100	100

A, amoxicillin; AZM, azithromycin; C, clarithromycin; ITT, intention to treat; LVFX, levofloxacin; mITT, modified ITT; MNZ, metronidazole; P, proton pump inhibitor; Seq, sequential therapy.

^1^ mITT: excludes patients who refused to start medication.

^2^ rPAC: PAC regimen with reduced dose of amoxicillin.

#### All Adverse Events

Seven studies reported adverse events, summarized by treatment regimen (Table 4). The adverse-event rate was highest (45%) with standard-dose PAC. Adverse rates with other regimens were lower than those for standard-dose PAC and were generally comparable across regimens, ranging from 0% to 22%. A detailed adverse-event summary is provided in the online supplementary Table 2.

**Table 4.** Adverse events in each group

Regimen	Patients (*n*)	Adverse events (*n*)	Adverse event rates (%)
PAC	75	34	45
rPAC^1^	166	16	9.6
MNZ	83	3	3.6
PA+AZM	44	3	7
PA+LVFX	22	0	0
PC	9	2	22
Seq	82	8	9.8

A, amoxicillin; AZM, azithromycin; C, clarithromycin; LVFX, levofloxacin; MNZ, metronidazole; P, proton pump inhibitor; Seq, sequential therapy.

^1^ rPAC: PAC regimen with reduced dose of amoxicillin.

### Additional Analysis

Three sensitivity analyses were performed: (a) eradication rates after excluding one study involving patients with chronic renal failure, (b) per-protocol eradication rates, and (c) eradication rates after excluding the single trial contributing to the PC arm [21]. All results aligned with the primary analysis (shown in online suppl. Figs. 3 and 4; league tables for sensitivity analyses (a) and (b) are provided in online supplementary Tables 3 and 4); analysis (c) did not materially change the estimates for the remaining comparisons.

## Discussion

This network meta-analysis aimed to integrate existing evidence on eradication in patients on HD, enabling selection of an efficient regimen for this population. The evidence is very uncertain about the effect of the PC regimen on the eradication rate relative to PAC. For the other regimens, the estimates were too imprecise to determine whether a difference from PAC exists (low certainty). Regarding safety, adverse events were most often reported with the standard-dose PAC regimen. Conversely, no substantial differences in adverse event rates were observed among the other treatment regimens. Therefore, reduced-dose PAC regimens, alternative regimens (with azithromycin, metronidazole, or levofloxacin), and sequential regimens may be considered for *H. pylori* eradication in HD patients, rather than standard-dose PAC or PC regimens.

To our knowledge, this is among the first network meta-analyses comparing *H. pylori* eradication regimens in patients undergoing HD. A recent review reported that eradication rates in patients on HD were comparable to those in non-dialysis populations, whereas standard-dose regimens were associated with increased adverse events; dose-reduced regimens may offer a safer alternative [2]. In that review, reduced-dose PAC regimens were compared with standard-dose PAC regimens, and alternative and sequential regimens were discussed narratively. Conversely, this network meta-analysis enabled quantitative comparison of alternative and sequential regimens relative to the standard-dose PAC regimen. Regarding adverse events, the present findings indicate that the standard-dose PAC regimen was associated with a higher frequency of adverse events, consistent with the review’s conclusion. Overall, multiple regimens seemed to achieve comparable eradication rates with better tolerability compared to standard-dose PAC, extending prior evidence focused primarily on dose reduction.

Because the estimates were too imprecise to determine whether differences in eradication rates exist among most regimens in patients receiving HD, regimen selection should be guided by tolerability and local feasibility, including regional antimicrobial resistance patterns and drug availability. In the general non-dialysis population, eradication success is influenced by antibiotic resistance rates, and current guidelines consistently recommend antimicrobial susceptibility testing to tailor therapy when feasible [9, 13, 14, 32, 35]. When the present findings are considered alongside current guidelines, one possible clinical framework is that reduced-dose PAC or sequential regimens containing clarithromycin may be considered where clarithromycin resistance is low or susceptibility is confirmed, whereas non-clarithromycin alternative regimens may warrant consideration where clarithromycin resistance is high or unknown, depending on local susceptibility patterns and drug availability. Nevertheless, patients receiving HD may have distinct safety considerations and medication constraints; therefore, these considerations should be applied with clinical judgment. When antimicrobial susceptibility testing is unavailable, current American College of Gastroenterology and European guidelines recommend bismuth-based quadruple therapy as empiric first-line treatment [13, 14], although this regimen was not evaluated in the present network. Local feasibility may further constrain treatment choice. In Japan, a clarithromycin-based regimen is currently approved and covered by national health insurance as first-line eradication therapy, whereas a metronidazole-containing regimen is approved as second-line therapy [9]. Given the low certainty of the evidence and the absence of region-stratified analyses, these considerations are contextual and should not be interpreted as treatment recommendations or a direct ranking of regimens.

This network meta-analysis has some limitations. First, the number of included studies was limited. Nonetheless, to address this limitation, evidence was carefully screened and extracted using a network meta-analysis with the CINeMA framework, following the Cochrane Handbook. Second, sub-analysis of potential effect modifiers by study region could not be performed because of the limited number of included studies. However, the influence of this factor may be limited because antimicrobial resistance rates for each antibiotic in this network are broadly comparable across regions. Third, nine of the ten trials were rated as having some concerns for risk of bias, mainly because the randomization process was incompletely reported and registered protocols or prespecified analysis plans were unavailable. Although domains related to deviations from intended interventions, missing outcome data, and outcome measurement were generally rated as low risk, the predominance of some-concerns judgments may reduce confidence in the network estimates by leaving residual uncertainty about selection bias and selective reporting. This limitation was incorporated into the within-study bias domain of CINeMA and contributed to the low-to-very-low certainty of the evidence. Finally, certainty of the evidence for the primary outcome was rated as low to very low, and results may change as further evidence accumulates. Therefore, additional studies are required to confirm the optimal *H. pylori* eradication regimen in patients undergoing HD.

In conclusion, reduced-dose PAC, alternative regimens containing azithromycin, metronidazole, or levofloxacin, and sequential regimens may be considered for *H. pylori* eradication in patients receiving HD. This cautious interpretation is based on the absence of clear differences in eradication-rate estimates across regimens other than PC and the relatively high adverse-event rate observed with standard-dose PAC. Further trials comparing reduced-dose PAC, alternative regimens containing azithromycin, metronidazole, or levofloxacin, and sequential regimens are required to determine the most appropriate *H. pylori* eradication regimen for HD patients.

## Statement of Ethics

In this network meta-analysis, no direct patient data were analyzed. A Statement of Ethics is not applicable because this study is based exclusively on published literature.

## Conflict of Interest Statement

Tomonori Yano was a member of the journal’s Editorial Board at the time of submission. This study was conducted in accordance with the guidelines developed by the Japanese Society of Gastroenterology.

## Funding Sources

No funding was received to assist with the preparation of this manuscript.

## Author Contributions

Conceptualization: Satoshi Sato, Jun Watanabe, and Takeshi Kanno. Methodology: Jun Watanabe. Formal analysis and investigation and writing – original draft preparation: Satoshi Sato and Jun Watanabe. Writing – review and editing: Keigo Misawa, Hiromi Sekiguchi, and Takeshi Kanno. Supervision: Tomonori Yano, Atsushi Masamune, and Tomoari Kamada.

## Data Availability

All data generated or analyzed during this study are included in this article and its online supplementary material files. Further inquiries can be directed to the corresponding author.
